# Case based teaching at the bed side versus in classroom for undergraduates and residents of pediatrics

**Published:** 2014-07

**Authors:** MAHDI SHAHRIARI

**Affiliations:** Department of Pediatrics, Hematology-Oncology Branch, Shiraz University of Medical Sciences, Shiraz, Iran

**Keywords:** Teaching, Lecture, Student, Pediatric, Resident

## Abstract

**Introduction: **Bedside teaching is defined as teaching in the presence of a patient, it is a vital component of medical education. The aim of this study was to evaluate the effectiveness of two methods of case based teaching (at the bedside and in the classroom) in the teaching hospitals (for both undergraduates and residents of pediatrics).

**Methods: **Thirty undergraduates and twenty pediatric residents were asked to study a topic of their curriculum from their text then pretest was taken from learners in the two levels; then either lecture with power point or case presentation or bed side discussion were conducted. One week later post- test was taken, and then evaluation of these three methods was done by a questionnaire from learners.

**Results: **The majority of under-graduates and all of pediatric residents had evaluated case based teaching superior to bedside teaching and these two methods superior to lecture method.

**Conclusions: **They believed that in the case based teaching they are more relaxed and have more self-esteem than at the bedside of the patients. Clinician teacher must involve participants and learners in the process of bedside teaching, by preparing a comfortable situation and by using available technology.

## Introduction

Bedside teaching is a vital component of medical education and one of the most effective ways to learn clinical and communication skills (1, 2). Bedside teaching is defined as teaching in the presence of a patient. Generally, it is thought that bedside teaching is applicable only to the hospital setting. However, bedsides teaching skills apply to any situation where the teaching occurs in the presence of a patient, including an office setting and long-term care facility (3, 4).

Sir William Osler (1849-1920), a renowned clinician-teacher, put emphasis on the importance of bedside teaching. In 1903 he stated “To study the phenomena of disease without books is to sail an uncharted sea, whilst to study books without patients is not to go to sea at all (5). To evaluate the effectiveness of two methods of case based teaching (at the bedside and in the classroom) in the teaching hospitals (for both undergraduates and residents of pediatrics) this study was conducted.

## Methods

From January 2011 to September 2013; in a pediatric hematology ward and clinic affiliated to Shiraz University of Medical Sciences with undergraduate and pediatric residents, this study was conducted for evaluation of efficacy of teaching methods: lecture, case based teaching, bedside teaching in these two levels. Thirty undergraduates and twenty pediatric residents were asked to study a topic of their curriculum from their text then pretest was taken from learners in the two levels; then either  lecture with power point or case presentation or bed side discussion were conducted. One week later post- test was taken, and then evaluation of these three methods was done by a questionnaire from learners. 

## Results

twenty eight (93.33%) of under-graduates and twenty (100%) of pediatric residents had evaluated case based teaching superior to bedside teaching and these two methods superior to lecture method . Comparison of pre-tests and post-tests showed better problem solving by learners after both case based teaching and bedside teaching methods: undergraduates and pediatric residents gained 80% better scores after bedside teaching; 83% better scores after case based teaching and 65% better scores after lecture. Main disadvantages of bedside teaching from learners’ point of view were false preceptors’ concern about patients’ comfort and misunderstanding in one side and becoming ashamed or disappointed when they had wrong answers or comments in the presence of the patients. They believed that in the case based teaching they are more relaxed and have more self-esteem than at the bedside of the patients. 

## Discussion

The bedside is valued as a site of learning from a real patient that is alive and tangible. It is, therefore, easier for learners to recall and remember the clinical situation (3, 4) and a clinician-teacher should allocate more time for it, which needs a detailed planning. This may add some time to that normally spent with the patient, but could provide a major experience. Students or residents who learn to solve a problem will learn both content knowledge and thinking strategies. A teacher in a clinical environment has a complex task, which can be described as; (a) an information provider, (b) a role model, (c) a facilitator, (d) an assessor; (e) a curriculum and course planner, and (f) a resource material creator. Many clinicians assume the task without adequate preparation or orientation (5, 6). By providing a chance for asking relevant question to obtain history and develop physical examination skills in a sympathetic manner, teaching at the bedside presents an excellent opportunity for the modeling of professional behaviors. It provides active learning in real context observes students' skills, increases learners' motivation and professional thinking, integrates clinical, communication, problem solving, decision making and ethical skills (1, 2, 6).

In this study although both levels of learners had better learning of the scheduled topic by both case based teaching and bedside teaching than lecture method, but they preferred case based teaching to bedside teaching due to reasons that are not as  strong as they can minimize  effectiveness of bedside teaching. Bedside teaching cannot be replaced with anything else. It has significant effect on patient care in the future practice of today’s students and residents.  There are newer approaches of effective bedside teaching, and the core focus of all such approaches is educational process.

## Conclusion

A bedside teacher must learn how to involve patients and learners in the educational processes. Moreover, bedside teaching is the process through which learners acquire the skills of communication by asking patients’ permission, establishing ground round rules, setting time limit, introducing the team, diagnosing learner, diagnosing patient, conducting focused teaching, using simple language, asking patient if there is any question, closing with encouraging thanks, and giving feedback privately. It is most important to ensure a comfortable environment for all participants, the learner, the patient and the bedside teacher.

In some situations problem based learning and case based learning may be mixed with bed side learning, to overcome known limitations of bedside teaching including time constraint, false preceptors’ concern about patients’ comfort and misunderstanding, short stay of patients in hospitals, learner distraction by technology (mobile and notebooks,…). A bedside teacher must learn how to involve patients and learners in the educational process. Maintaining a comfortable environment for all participants; the learner, the patient and the bedside teacher is very important. It is through this process that the learners acquire the skills of observation, communication, examination and professionalism. 
